# There and back again: from the origin of life to single molecules

**DOI:** 10.1007/s00249-018-1295-1

**Published:** 2018-03-22

**Authors:** Petra Schwille

**Affiliations:** Max Planck Institute of Biochemistry, Am Klopferspitz 18, 82152 Martinsried, Germany

**Keywords:** Fluorescence correlation spectroscopy, Single molecule detection, Molecular evolution, Synthetic biology

## Abstract

What is life? There is hardly a more fundamental question raised by aspiring researchers, and one less prone to ever be answered in a scientifically satisfying way. In the long, productive and highly influential period of research following his Nobel-recognised work on relaxation kinetics, Manfred Eigen made seminal contributions towards a quantifiable definition of life, with a strong focus on its evolutionary character. In the last years of his time as an active researcher, however, he devoted himself to another, purely experimental topic: the detection and analysis of single biomolecules in aqueous solution. In this short review, I will give an overview of the groundbreaking contributions to the field of single molecule research made by Eigen and coworkers, and show that both, in its intrinsic motivation, and in its consequences, single molecule research strongly relates to the question of the physical–chemical essence of life. In fact, research on living systems with single molecule sensitivity will always refer the researcher to the question of the simplest possible representation, and thus the origin, of any biological phenomenon.

## Introduction

In 1994, a review paper appeared that greatly inspired a generation of researchers at the interface of chemistry, physics, and biology. To date, it has been cited more than 1000 times, and a wealth of research projects, patents, and even companies have followed in its wake. “Sorting single molecules: Application to diagnostics and evolutionary biotechnology” (Eigen and Rigler 1994) is a hallmark of scientific communication co-authored by National Academy member Manfred Eigen, a grand scientific authority and Nobel laureate. Remarkably, in spite of his many outstanding contributions to science, this late one turned out to become his third highest cited journal paper. Even more remarkable, however, is its scientific content, which is only moderately review-like in character, instead rather serving the purpose of a visionary perspective. If submitted by less eminent authors, editors and reviewers would have potentially requested to straighten out this obvious discrepancy, but the scientific community would have suffered a great loss if it had not appeared as such. Briefly, in this paper, the authors express their excitement about a new technical breakthrough that Rigler and his coworkers had previously accomplished: the direct detection of single fluorescently labeled molecules diffusing freely in aqueous solution.

That this has become possible was primarily due to three important accomplishments, two of them being purely technical—the availability of stable and sufficiently strong lasers on one hand, and of sensitive-enough detectors to record single photons on the other hand. The most important and ingenious accomplishment, however, was the optical setup of the new single molecule device presented in the framework of a method called FCS—Fluorescence Correlation Spectroscopy. It consisted of a confocal optical pathway, in which the laser was focused down to the resolution limit by an objective with high numerical aperture (NA), and the focal spot so created projected through an aperture with similarly small orifice in the image plane, a so-called pinhole, which limited the detection volume additionally in axial dimension. This produced open volume elements in the femtoliter range—nine orders of magnitude below what could be pipetted in the lab, and close to the size of single bacterial cells. FCS was at that time already a twenty-year-old concept, originally designed as a technique to analyze spontaneous fluctuations around a thermodynamic equilibrium—and thus, in fact, a relaxation technique such as the ones that Eigen had pioneered, but with the important difference that no external disturbances needed to be induced, such as a pressure or temperature jump (Magde et al. 1972; Ehrenberg and Rigler 1974). The history of how FCS was originally conceived by the consequent extrapolation of Eigen’s own methods is nicely set out in another article in this issue (Rigler and Widengren, this issue). To make FCS work, however, the ability to resolve random thermodynamic fluctuations on the molecular level was indispensable, and this first became convincingly possible by the new confocal illumination and detection (Rigler and Widengren 1990; Rigler et al. 1993). Once being able to detect the presence of single molecules, and their fluctuating number in an open volume element, through random bursts of fluorescence, temporal autocorrelation analysis yields statistically relevant information about all kinds of processes that influence the dynamics of these molecules, from simple three-dimensional diffusion to inter- and intramolecular reactions.

The presentation of FCS and the breakthrough accomplishment of its confocal representation by Rigler and his colleagues to a broader audience was, however, only one motivation of the 1994 PNAS paper. Another one was the implication that single molecule detection obviously had to the other big scientific topic of Eigen’s research: evolutionary biotechnology. The recognition that evolutionary mechanisms manifest themselves at much simpler levels than the ones of living organisms, which Eigen had greatly supported by his quantitative concepts, such as the molecular quasispecies theory, made it extremely exciting to speculate about the possibility of directly evolving molecules by rounds of mutation and selection, i.e., without having to go through the tedious process of amplifying them by the genotype–phenotype coupling of producing organisms. Being able to observe functional differences in single molecules, and to sort them according to these differences, would open up the possibility for the researcher to artificially establish any kind of fitness measure for selection that he/she would have in mind. Besides that, a detection sensitivity that high would have exciting consequences for biomedicine in a much broader sense: the possibility to detect traces of infectious agents, such as single viruses, in blood serum, would open up a completely new chapter of diagnostics. The last vision that Eigen and Rigler brought forward in this seminal article sounded even more far-fetched: the possibility of single molecule DNA sequencing, based on the successive detection of fluorescently labeled single bases or base-pairs.

Of course, the ability to optically detect the transits of single fluorescent molecules through the light cone of a laser focused into an aqueous environment, although a technical breakthrough at that time, was still a long stretch away from the ability of actually identifying them individually, or even of finding them in a volume nine or more orders of magnitude larger than the confocal probe volume. With respect to evolutionary biotechnology, the challenge was in fact even greater, as detection was only the first step towards any possible cycle of variation, selection and amplification. How to hold on to a single molecule in solution? Although, intriguingly, the following decades indeed brought forward practical realizations of molecular traps (Fields and Cohen 2010), Eigen and Rigler already then sketched some solutions, based on a combination of microfluidic sample handling and electrical fields, that were remarkably visionary. Microsystems technology had at that time just started to provide attractive miniaturization perspectives for the biosciences. Indeed, only a couple of years after the release of this article, researchers accomplished the detection and tracking of single fluorescent molecules (Schmidt et al. 1996), and astonishingly, there are now even commercial solutions for single molecule DNA sequencing (Eid et al. 2009). However, the original idea of sorting single molecules for evolutionary biotechnology had been dropped by Eigen and Rigler after a relatively short time, due to the many obvious experimental difficulties presented, and has to my knowledge not been followed up on by other groups to date. Instead, Eigen and coworkers pioneered many other influential applications in the following decade, with a strong focus on molecular diagnostics, which I will briefly review in the next section.

## Applications and variations of FCS in the 1990s

In the years following the 1994 PNAS paper, there were two technical breakthroughs in particular that greatly helped FCS to receive the attention that Eigen and Rigler had envisioned. This was the combination of FCS detection with microfluidic sample handling on one side (Brinkmeier et al. 1999; Dörre et al. 1997), and the establishment of dual-color cross-correlation on the other side (Schwille et al. 1997). Playing to the strengths of the rising microsystems technology, advocating the “lab on the chip”, it became possible to sample volumes much larger than the confocal spot in a relatively short time, and by this overcome the limits of passive diffusion, which is fast on small scales, but painstakingly slow on large scales. The theory of fluctuation correlation analysis of flowing samples (Magde et al. 1978) was expanded, and it could be shown that by implementing spatial correlation between two or more different volume elements, flow directions could be determined (Brinkmeier et al. 1999), and calibration-free diffusion measurements without knowing the exact shape and size of the measurement volumes were possible (Dittrich and Schwille 2002).

The impact of dual-color cross-correlation spectroscopy (FCCS) realized by Schwille et al. (1997) received even more widespread attention, as it provided, for the first time, a direct way to measure molecular interactions between two species of molecules in any transparent environment, and with single molecule sensitivity. In contrast to the analysis of irreversible reactions through the change of diffusion of one, usually small, reaction partner upon binding to a large or immobile target, which had up to then been the most prominent FCS application to biochemical kinetics (Kinjo and Rigler 1995), FCCS now induced no more constraints to molecular sizes. First demonstrated with a differently labeled pair of complementary oligonucleotides and later expanded to protein–protein interactions in binary or even ternary systems (Heinze et al. 2004), FCCS has been established as a very powerful technical advance to FCS that later even found commercial realization. In FCCS, the shape of the cross-correlation curve compiled from two separate fluctuating fluorescence signals, resulting from different species of molecules diffusing through a focal spot illuminated by two different laser beams, is no longer the primary source of information. Instead, the focus of FCCS analysis is on the amplitude of the curve, reflecting directly on the presence of dual-colored molecules, usually the reaction product from two species of single-colored species, supposed to interact with each other. However, also the reverse reaction, the cleavage or breaking of bonds between two molecules, or two subunits of a single molecule, each bearing a separate fluorescent label, can be precisely quantified by FCCS. This could be demonstrated by the analysis of enzymatic cleavage reactions, and thus, specifically, the activity of endonucleases and proteases on double-labeled DNA templates or proteins (Kettling et al. 1998; Kohl et al. 2005).

With respect to the diagnostic potential of FCS, exciting applications with fluorescently labeled short DNA primers targeted to large DNA or RNA targets from pathogens, such as HIV, could be demonstrated (Walter et al. 1996; Oehlenschläger et al. 1996). Already small traces of pathogenic DNA, when amplified by PCR, resulted in a noticeable difference in the average diffusion behavior of small probes, evidenced by a shift of the autocorrelation function to larger decay times. The kinetics of these recognition reactions depended critically on the number of corresponding accessible base-pairs and could thus even be used to assess secondary structures of targets (Schwille et al. 1996). Also, the onset of probe elongation allowed the total number of pathogenic nucleic acid molecules to be inferred, and positive samples to be distinguished from false positives.

In the mid-90s, another alarming class of pathogens had just started to surface: the prions—infectious protein particles, supposed to induce the misfolding, aggregation and cytotoxicity of cellular proteins, resulting in severe damage and degeneration of neurons in the brain, and in fatal diseases such as “Mad Cow” and Creutzfeldt-Jakob. Due to the excellent features of FCS to access protein aggregation through the change of molecular brightness and diffusion, which had been outlined very early as a key application (Meyer and Schindler 1988), prion protein diagnostics became the latest but definitely not least successful line of active research in Manfred Eigen’s department. Initiated by a theoretical article about the potential mechanisms of infectivity on the molecular level (Eigen 1996), the group combined FCS and particularly FCCS with strategies for large sample handling: identifying rare species of early infectious aggregates in a large background of soluble monomers was apparently the greatest challenge for diagnostics. Small traces of double-colored early aggregates having been formed by differently labeled monomeric precursors could be best identified by scanning the laser focus through the sample, or by flowing the sample through microfluidic channels crossing the detection volume (Bieschke et al. 2000).

In these dual-color applications, it became increasingly evident that the mathematical correlation procedure which gave FCS and FCCS its name was not really relevant anymore. Rare events, such as the occurrence of aggregates or of other sparse infectious targets could be evidenced through the simultaneous occurrence of fluorescence bursts, a so-called coincidence, in two separate measurement channels. This recognition was also the starting point to the late truly biotechnological applications of FCS-related methods in the Eigen group. They continued with the concept of characterizing enzymatic activity through the formation or breakage of bonds in target molecules, but now optimized the performance of the method towards true large number screening (Koltermann et al. 1998; Winkler et al. 1999; Heinze et al. 2002). Scanning the beam or flowing the sample, in addition to more quickly helping to find rare species like needles in a haystack, also greatly helped to decrease the time required to compile statistically relevant signals from diluted solutions. To analyze enzymatic activity, the perspective of recording meaningful data from very small amounts of enzyme and template in a short amount of time laid the foundation for screening routines, where thousands of different types of enzymes, e.g., evolutionary optimized ones, could be tested in fractions of a second each. Thus, this late streak of applications published by the Eigen group to some extent confirmed the visions laid out in the 1994 PNAS paper, although the direct detection and sorting of evolved molecules themselves was no longer a goal, but all the more the smart adaptation of FCS-related routines for commercial applications.

## Continuation towards cells and organisms in the 2000s

With respect to basic science, it soon appeared that FCS and FCCS, realized in a very similar instrumental setup as used for confocal laser scanning microscopes, which now became very widespread in the biosciences, would be a fantastic complement to imaging for quantitatively targeting processes in live cells or even organisms. The very fundamental information about fluorescently labeled molecules that FCS could deliver—concentrations and mobilities, turned out to be important for the characterization of biochemical processes occurring in living cells. In the postgenomic era, the new challenge of proteomics has been to yield detailed information about the amount of expressed proteins in any cell, as well as their multiple interactions. To access these data separation-free in an unperturbed system has been a great promise by FCS. Thus, cellular applications of FCS and FCCS became increasingly popular in the 2000s, aided by the commercial instrumental solutions that were often integrated into laser scanning microscopes (Schwille 2001). Early cellular FCS yielded diffusion constants in the cytoplasm, the nucleus and the membrane (Brock et al. 1998; Schwille et al. 1999a, b; Wachsmuth et al. 2000), as well as transport processes in tubular structures (Köhler et al. 2000; Gennerich and Schild 2000). With regard to molecular interactions, dual-color FCCS allowed the determination of cellular enzyme activity (Kohl et al. 2005), to characterize various mechanisms of endocytosis (Bacia et al. 2002), and to study protein–protein interactions to the point that even reactions with variable stoichiometry could be quantified (Kim et al. 2005, 2007).

However, it soon became apparent that there are two major practical challenges which render FCS measurements in cells particularly complicated, or even preclude them: The first one is photobleaching, which always occurs at high light intensities as used for confocal illumination. It is a nuisance for all fluorescence applications, including imaging, but particularly harmful for FCS, as it introduces additional time-scales to the dynamic analysis. The second challenge is more fundamental in nature. The theoretical concept of FCS is targeted towards analyzing fluctuations around thermodynamic equilibrium. The physiological situation in cells, however, is far away from being equilibrated. In addition to that, the requirement of a small open probe volume in a much larger sample pool is often not fulfilled, due to the cellular ultrastructure, retaining many molecules in compartments close to or even far below the resolution limit. This requires very careful calibration of the optical setup and preparation of the biological system, as well as the use of smart routines for data processing.

It has been demonstrated that the cumulative photobleaching due to small reservoirs in cells can be dramatically reduced by employing fluorescence two-photon excitation (Schwille et al. 1999a, b). Herein, the excitation laser is tuned to approximately twice the absorption wavelength of the respective dyes. Using pulsed excitation with high photon flux during the pulses, there is a significant probability of simultaneous absorption of two photons, which add their energy to make the required transition to the excited state of the fluorophore. The resulting square dependence of fluorescence on intensity shows a very strong spatial decay away from the focal plane, significantly reducing out-of-focus photobleaching. In addition, the much broader excitation spectra of standard dyes for two-photon excitation allow the simultaneous excitation of spectrally distinct labels, as used for dual-color FCCS, with one beam (Heinze et al. 2000; Kim et al. 2004).

Another instrumental solution to minimize dynamic and cumulative photobleaching is to use beam-scanning, which had already been successfully applied in the diagnostic applications outlined above. The scan pathway can either be a closed circle sampled at constant scan speed, enabling data recording throughout the scan (Petrásek and Schwille 2008), or a straight line, which is scanned repeatedly. Here, due to the requirements of turning around, constant recording cannot be carried out, and smart data processing has to be applied to identify and separate the right signal periods (Ries et al. 2009a, b). Utilizing these sophisticated scanning and data recording procedures, FCS was enabled in cells and organisms with optically quite precluding conditions, such as bacteria and yeast, as well as *C. elegans* and *D. rerio* embryos (Meacci et al. 2006; Petrásek et al. 2008; Ries et al. 2009a, b). FCCS in early zebrafish embryos allowed the quantitative mapping of the establishment of morphogen gradients (Yu et al. 2009) and quantification of the affinity of receptor–morphogen interactions in situ (Ries et al. 2009a, b).

## Towards minimal biological systems

In spite of the great success stories of FCS and related single molecule techniques when applied to cells, it became in the last years even more evident that such sensitive methods will be subject to all kinds of nonidealities and disturbances occurring in living systems. Parameters that can be determined with very high statistical accuracy in well-defined solutions will suffer from great tolerances and huge error bars when measured in the much less well-defined and controlled cell interior. Even worse, it will hardly be possible to unequivocally assign the effect of any change in condition on a cellular process to a particular variable, as indirect effects or the involvement of unknown other factors can never be fully ruled out. Thus, meaningful cellular applications of FCS have increasingly been complemented by applications in reconstituted systems, retaining the essential features and variables, but otherwise greatly simplifying the landscape of relevant parameters. The most popular platform for such cell mimetic studies are giant unilamellar vesicles (GUVs) that provide a perfect environment for investigating processes taking place on cell membranes (Korlach et al. 1999; Schwille et al. 1999a, b). They have been particularly influential in characterizing the effect of local membrane structure and fluidity on the diffusion and interactions of lipids and proteins (Bacia et al. 2004; Kahya and Schwille 2006). In these greatly simplified systems, it became apparent that physical phenomena, such as lipid phase separation, or mechanical constraints due to cytoskeletal filaments attached (Heinemann et al. 2013) influence key biochemical processes and thus, dynamics as measured by FCS.

Reconstitution of biological functions in minimal systems, i.e., systems with a minimal number of species and/or free parameters, has generally become very attractive also for investigations by single molecule imaging. The most prominent scenario is the analysis of single molecules of motor proteins, e.g., when moving along filaments (Vale et al. 1996). It has led to new mechanistic insight or confirmed existing hypotheses, such as the recognition of rotary movement of the ATP synthase through an attached actin filament (Noji et al. 1997). For biophysics in general, the concept of assembling biological functionality from the bottom-up, in a synthetic approach, seems very appropriate and rewarding when aiming for reproducible and quantitatively meaningful results (Schwille and Diez 2009). On the other hand, this can only be achieved by at least partly sacrificing the “physiological relevance” as cherished by most biologists. That leads to a certain dichotomy, nowadays observed with respect to the understanding of biology. Do we understand biology by taking into account each and every aspect of its compositional and dynamic makeup? Or do we understand biology by trying to separate underlying fundamental principles from specific, presumably exchangeable, compositional representations? In other words: is today’s obvious physiological complexity an attribute of life that was acquired through evolution, or is it the essence of life as we know it? If the former is true, it is exciting to speculate about greatly simplified models of living entities, which would be the ideal study objects for all biophysical techniques, but first and foremost for the exquisitely sensitive single molecule methods.

## Conclusions and outlook: back to the origin of life

Intriguingly, this leads us back to the question about the nature and origin of life that had obviously also motivated Eigen’s work on molecular evolution. Has life started simple, and if so, could we reconstitute a biochemical system that indeed allows to witness a transition from chemistry into biology? In particular, which ones would be the first constitutive processes? Eigen mainly focused on the emergence of information and, in particular, of evolutionary mechanisms, but there are other fundamental features, such as metabolism and compartmentation, whose functional origins also deserve great attention (Schwille 2017). From our own recent work on the emergence of self-organization and pattern formation in minimal protein–membrane systems (Loose et al. 2008; Zieske and Schwille 2014), we can conclude that single molecule methods such as FCS are uniquely suited to characterize and understand these fundamental processes. This now finally closes a circle: single molecule analysis had been proposed by Eigen and Rigler to aid the understanding of molecular evolution. And after many years of single molecule research, we are referred back to the initial questions of the origin of biology, when searching for the optimal system to apply our single molecule methods to.
